# Coupling day length data and genomic prediction tools for predicting time-related traits under complex scenarios

**DOI:** 10.1038/s41598-020-70267-9

**Published:** 2020-08-07

**Authors:** Diego Jarquin, Hiromi Kajiya-Kanegae, Chen Taishen, Shiori Yabe, Reyna Persa, Jianming Yu, Hiroshi Nakagawa, Masanori Yamasaki, Hiroyoshi Iwata

**Affiliations:** 1 Department of Agronomy and Horticulture, University of NE-Lincoln, Lincoln, NE 68583 USA; 2 Graduate School of Agricultural and Life Sciences, The University of Tokyo, Bunkyo, Tokyo, 113-8657 Japan; 3 Institute of Crop Science, National Agriculture and Food Research Organization (NARO), Tsukuba, Ibaraki 305-8518 Japan; 4 Department of Agronomy, Iowa State University, Ames, USA; 5 Food Resources Education and Research Center, Graduate School of Agricultural Science, Kobe University, Kasai, Hyogo 675-2103 Japan; 6 Institute for Agro-Environmental Sciences, National Agriculture and Food Research Organization (NARO), Tsukuba, Ibaraki 305-8604 Japan

**Keywords:** Agricultural genetics, Genetic interaction, Genomics, Plant breeding, Plant genetics, Computational biology and bioinformatics, Genetics, Plant sciences, Astronomy and planetary science, Mathematics and computing, Physics

## Abstract

Genomic selection (GS) has proven to be an efficient tool for predicting crop-rank performance of untested genotypes; however, when the traits have intermediate optima (phenology stages), this implementation might not be the most convenient. GS might deliver high-rank correlations but incurring in serious bias. Days to heading (DTH) is a crucial development stage in rice for regional adaptability with a significant impact on yield potential. The objective of this research consisted in develop a novel method that accurately predicts time-related traits such as DTH in unobserved environments. For this, we propose an implementation that incorporates day length information (DL) in the prediction process for two relevant scenarios: CV0, predicting tested genotypes in unobserved environments (C method); and CV00, predicting untested genotypes in unobserved environments (CB method). The use of DL has advantages over weather data since it can be determined in advance just by knowing the location and planting date. The proposed methods showed that DL information significantly helps to improve the predictive ability of DTH in unobserved environments. Under CV0, the C method returned a root-mean-square error (RMSE) of 3.9 days, a Pearson correlation (PC) of 0.98 and the differences between the predicted and observed environmental means (EMD) ranged between -4.95 and 4.67 days. For CV00, the CB method returned an RMSE of 7.3 days, a PC of 0.93 and the EMD ranged between -6.4 and 4.1 days while the conventional GS implementation produced an RMSE of 18.1 days, a PC of 0.41 and the EMD ranged between -31.5 and 28.7 days.

## Introduction

To satisfy the daily food demands of a growing population, it is necessary to improve the crop production systems to allow a sustainable increase of the yield potential and nutritional value of the new and up-coming varieties^1^. In plant breeding there are different tools and methodologies to increase the rate of genetic gain^2^. Traditional plant breeding methods use phenotypic and/or pedigree information to perform selection of experimental lines for developing superior breeding lines. However, phenotypic selection may not be permissible in many situations due to the high phenotyping costs and limited availability of resources (land, water, seed availability, etc.); also, pedigree selection may not be accurate since it assumes a constant rate of recombination (50% of the genetic material coming from mother and the remaining 50% coming from father) and this might not be a feasible premise. In this regard, the use of molecular markers can help to detect the deviations from the expected genetic material of the parents and can be used to determine which parental haplotypes were inherited.

Genomic selection^3^ (GS) is an emerging tool that allows screening genotypes from a very large population without having to observe them in fields^4,5^. This method only requires phenotypic and genomic information for calibrating models, then other genotyped candidates are selected based on the predicted genetic values obtained via their marker profiles^6^. In this context, the implementation of genomic tools and resources translates our understanding of the relationship between genotype and phenotype into predicted genetic merits for direct selection. This is highly desirable when dealing with those traits that are controlled by a large number of genes with small individual effects, also known as complex traits^5,7^. GS has shown to be an effective tool for plant and animal breeding applications, especially for complex traits^8^. The success of GS predicting phenotypes from genotypic information has been reported in various crop species^9^.

In GS, the genomic estimated breeding values (GEBVs) of complex traits of untested genotypes are computed based on genome-wide marker information. The GEBVs can be considered as deviations around a fixed mean (e.g., zero). Once the GEBVs are obtained, the breeders may conduct selections based on ranks by selecting those individuals in the tails. Depending on the trait, the individuals are selected either on the upper or lower tails of the ordered GEBVs. For example, the upper side is selected for increasing genetic gains of traits like grain yield, stress resistance, or the content of essential nutrients. On the other hand, genotypes in the lower tail are selected for reducing traits such as lodging, shattering, the content of anti-nutrients, etc. In these cases, the GEBVs provide sufficient information when the GEBVs and the direct genetic values (or phenotypic values) exhibit a high correlation. However, the rank-based selection criteria might not be the most accurate when the target trait has an intermediate optima. This is the case of those traits related with phenological stages of crop plants. For example, Onogi et al.^10^, studied the prediction of heading date of untested genotypes within and across environments by integrating genomic prediction with crop/ecophysiological modelling. These authors predicted DTH of untested genotypes in observed and unobserved environments among other cross-validations schemes. In their case, the obtained root-mean-square error (RMSE) for predicting untested genotypes in unobserved environments was almost twice as much as when predicting observed environments. Moreover, in both cases, high correlations between predicted and observed values were obtained. Predicting untested genotypes in observed environments the correlation was of 0.96 while for unobserved environments it was of 0.87. These results highlight the importance of improving the accuracy of predicting untested genotypes in unobserved environments despite the fact of obtaining high correlations.

In rice, days to heading (DTH; typically defined as the number of days after planting when more than 50% of plants show the heading stage) is one of the critical traits of ideotypes with an increased yield potential. Hence, it is important to optimize DTH for high yield potential in different environments^11^. Usually, multi-environment trials (METs) are performed for developing stable cultivars across a wide range of environmental conditions. Usually, breeders are interested in estimating DTH for a large set of environments instead of just a single one. However, when the GEBVs are computed for unobserved environments, these might contain a large bias if the phenotypic information from those genotypes in the training set does not resemble the target environment. The GEBVs from one environment may not be suitable for another due to variations in the environmental *stimuli*. This might cause inconsistencies in the response patters as result of the interaction between genotypes and environments (G × E interaction). These differences also remain for same genotypes observed at the same location but tested at different times (planting date, years). As consequence of the aforementioned issues, the problem of correctly estimating time-related traits such as DTH for a set of untested genotypes in a wide range of unobserved environments becomes harder to solve than for those traits where the selection is rank-based (e.g., yield). As pointed before, one of the advantages of GS techniques over conventional phenotypic methods is its ability for performing accurate rank predictions of untested genotypes based exclusively on their marker profiles. Thus, it is not necessary to observe these in fields in order to select the superior genotypes. Thus, a method capable of delivering highly accurate predictions of untested genotypes in unobserved environments without incurring a large bias is desirable.

As was pointed before, DTH in rice is important for regional adaptability and has usually great impact on yield potential^11^. The genetic systems controlling DTH have been studied for a long time by integrating Mendelian genetics and genomics^12^. The potential of GS in DTH has been evaluated in rice^13,14^ and sorghum^15^. Onogi et al*.*^10^ focused on the genomic prediction of DTH in multiple environments and demonstrated the potential of a novel method that integrates GS with a crop growth model. Li et al.^15^ also focused on the genomic prediction of DTH in multiple environments and showed the potential of combining GS with joint regression analysis on photothermal time. To our knowledge, no studies combining DL on the day when genotypes reach DTH with GS tools have been presented yet. The objective of this research was to develop a method for predicting time-related traits such as DTH in unobserved environments combining day length and molecular markers.

Two cross-validation schemes of interest for designing and planning experiments were considered for evaluating the performance of the proposed implementation for predicting DTH in unobserved environments: CV0, predicting already tested genotypes in unobserved environments (targeting environments); and CV00, predicting untested genotypes in unobserved environments (targeting genotypes and environments). For assessing the proposed implementation, the root-mean-square error (RMSE), the Pearson correlation (PC) and the difference between predicted, and observed environmental means (EMD) values were computed and compared with the results obtained from traditional GS models.

In this study, we propose two novel methods for predicting DTH of tested (curve’s method: C method) and untested (combining the curve’s method and the traditional genomic BLUP modeling: CB method) genotypes in unobserved environments in a precise and accurate way (i.e., small bias and high correlation between predicted and observed values).

Under the hardest cross-validation scheme (i.e., CV00), the results of the introduced CB method are compared with those obtained with the conventional GS implementation (G-BLUP). For studying the realistic scenario of predicting already tested genotypes in unobserved environments, the cross-validation scheme CV0 was considered. These results are used for assessing the levels of predictability that can be reached when the phenotypic information of a genotype is available in other environments. Here, the environments are considered “target” in a strict sense because these can be selected even before conducting the field experiments using the theoretical DL values of specific locations for a given planting date.

## Results

### Descriptive statistics

The box-plot of DTH is depicted in Supplementary Fig. S1. The environments were ordered based on their environmental medians. In general, those environments with late planting dates appear first. The medians ranged between 59 (“Fukuyama 2010 Late”) and 116 (“Akita 2015”) days. The within environments SDs varied between 5.07 and 16.83 days. Also, the smallest and the largest DTH values were 42 and 148 days and these were observed in “Tsukubamirai 2016 Late” and “Akita 2015” environments, respectively.

### Evaluating the bias and accuracy of the methods

Table 1 contains the observed and predicted environmental means (E-mean) for two different cross-validation schemes (CV00 and CV0) as well as the corresponding RMSE and the PC between predicted and observed values within environments. For the CV00 scheme, both the CB and GS methods were implemented. The objective of using this cross-validation scheme was to contrast the performance of the proposed method with respect to the conventional GS implementation for predicting untested genotypes in unobserved environments. Since we lack of phenotypic information for connecting yet to predict genotypes in unobserved environments with those genotypes in training sets, the CV00 scheme is considered the hardest and the most interesting prediction problem. Across environments, the conventional GS method returned an RMSE of 18.1 days and a PC of 0.41; within environments, the average RMSE and PC were of 17.5 days and 0.69. The EMD ranged between -31.5 and 28.7 days. On the other hand, across environments the proposed CB method returned an RMSE of 7.3 days and a PC of 0.93; within environments, the average RMSE and PC were of 7.2 days and 0.86. The EMD ranged between -6.4 and 4.1 days.

**Table 1:** Environmental means, Root Mean Squared Error (RMSE) and the Pearson correlation between predicted and observed days to heading (DTH) for 112 rice genotypes tested in 51 environments in Japan.

	CV00							CV0		
	Mean			RMSE		Corr		Mean	RMSE	Corr
	Real	CB	GS	CB	GS	CB	GS	C	C	C
Tsukubamirai 2004 Late	79.4	76.7	85.9	7.0	12.6	0.86	0.70	76.3	4.6	0.99
Tsukubamirai 2004 Early	87.5	93.2	84.1	10.0	12.9	0.84	0.55	92.2	6.4	0.97
Tsukubamirai 2005 Early	98.5	102.1	87.1	6.9	14.0	0.87	0.75	101.0	3.5	0.98
Tsukubamirai 2005 Late	76.7	74.8	84.8	5.7	12.4	0.86	0.68	73.7	3.4	0.99
Kasai 2006	87.4	83.8	88.3	7.9	8.8	0.85	0.75	82.8	6.8	0.93
Fukuyama 2006	80.6	83.0	82.5	5.6	13.4	0.87	0.29	82.0	2.0	0.99
Fukuyama 2007	84.1	83.4	86.6	5.0	6.1	0.87	0.84	82.5	2.9	0.98
Tsukuba 2008	104.5	107.7	87.2	9.1	19.2	0.87	0.85	107.3	4.0	0.98
Kasai1st 2008	102.2	99.9	88.7	9.0	17.8	0.89	0.71	99.2	4.3	0.99
Fukuyama 2008	77.8	82.2	85.6	7.8	11.1	0.86	0.77	81.3	4.5	0.98
Tsukuba 2009	103.9	107.7	87.1	8.6	19.0	0.89	0.81	108.0	5.2	0.98
Kasai1st 2009	101.9	100.6	89.4	7.6	15.1	0.89	0.83	100.0	2.7	0.99
Fukuyama 2009	78.7	78.7	89.6	5.2	13.2	0.86	0.83	77.8	1.5	0.99
Tsukuba 2010 Late	83.5	89.1	88.9	8.4	11.1	0.86	0.66	88.4	5.9	0.97
Kasai1st 2010	100.6	100.7	89.5	7.1	14.3	0.88	0.77	100.1	1.9	0.99
Fukuyama 2010	76.2	79.5	88.2	5.9	15.3	0.86	0.50	78.1	2.6	0.99
Fukuyama 2010 Late	59.0	65.4	87.7	9.2	29.7	0.84	0.82	62.5	4.2	0.96
Tsukuba 2011 Early	105.9	109.9	85.5	9.9	23.9	0.87	0.66	109.9	5.3	0.98
Tsukuba 2011 Late	82.8	80.8	90.0	5.9	11.9	0.86	0.78	80.4	4.1	0.95
Tsukuba 2011 Middle	94.7	95.7	90.9	6.5	11.1	0.88	0.67	95.4	3.0	0.98
Kasai 2011	101.4	100.6	89.3	7.8	15.7	0.89	0.76	100.0	2.4	0.99
Fukuyama 2011	75.7	79.4	91.1	6.4	16.9	0.87	0.83	78.6	3.1	0.99
Fukuyama 2011 Late	66.4	72.7	97.9	8.9	32.4	0.68	0.58	66.2	2.9	0.95
Tsukuba 2012 Early	97.1	96.0	86.9	5.7	12.4	0.81	0.74	101.1	5.8	0.97
Tsukuba 2012 Late	84.3	81.6	88.2	6.1	7.5	0.86	0.80	81.0	4.3	0.96
Tsukuba 2012 Middle	92.6	95.4	88.4	6.3	8.3	0.87	0.82	94.8	3.7	0.97
Kasai 2012	103.8	102.7	86.4	7.9	21.3	0.89	0.60	102.1	2.7	0.99
Fukuyama 2012	75.2	78.8	89.9	6.2	16.3	0.87	0.83	78.0	3.1	0.99
Fukuyama 2012 Late	71.5	77.0	103.1	7.5	32.4	0.70	0.66	68.6	3.4	0.98
Fukuoka 2012	79.6	76.0	89.1	6.7	13.0	0.84	0.69	75.4	5.2	0.96
Akita 2012	113.1	115.9	90.1	8.3	26.1	0.86	0.54	114.5	2.8	0.98
Kasai 2013	104.8	104.1	87.3	8.0	20.1	0.90	0.80	103.5	3.2	0.98
Fukuyama 2013	74.9	80.1	89.4	7.4	16.3	0.87	0.82	79.3	4.6	0.99
Fukuyama 2013 Late	62.1	64.8	85.7	6.3	27.1	0.83	0.36	64.1	3.6	0.93
Fukuoka 2013	77.4	77.3	93.3	5.7	17.5	0.86	0.77	76.7	1.9	0.99
Akita 2013	116.6	115.8	91.1	8.5	28.1	0.88	0.66	115.1	2.4	0.99
Kasai 2014	102.4	105.1	88.9	8.4	18.0	0.90	0.66	104.4	2.8	0.99
Fukuyama 2014	79.6	82.1	87.9	5.9	12.1	0.88	0.68	81.3	2.1	0.99
Fukuyama 2014 Late	67.6	64.5	88.1	6.7	21.4	0.83	0.79	62.9	5.1	0.98
Fukuoka 2014	81.8	77.7	80.4	6.7	11.5	0.87	0.45	77.1	5.4	0.97
Akita 2014	115.2	116.2	89.6	9.2	28.5	0.88	0.68	115.6	2.2	0.99
Fukuyama 2015	76.3	72.6	88.1	6.5	14.4	0.86	0.63	71.9	4.6	0.99
Akita 2015	118.1	119.8	89.4	7.7	29.9	0.81	0.76	117.6	2.5	0.99
Kasai 2016	102.3	101.2	85.6	7.2	20.4	0.90	0.61	100.7	3.1	0.98
Fukuyama 2016	79.1	81.1	85.2	6.0	8.9	0.86	0.82	80.8	2.4	0.99
Fukuyama 2016 Late	67.5	65.2	89.6	6.8	26.1	0.79	0.63	64.6	4.5	0.95
Tsukubamirai 2016 Late	73.6	77.9	84.6	7.0	13.6	0.90	0.80	77.2	4.9	0.98
Kasai 2017	108.6	106.2	85.7	8.3	26.3	0.89	0.56	105.5	4.3	0.98
Fukuyama 2017	77.4	80.2	87.7	6.3	16.3	0.88	0.42	79.6	2.7	0.99
Fukuyama 2017 Late	68.4	67.7	88.8	6.0	22.9	0.82	0.69	67.0	3.0	0.97
Tsukubamirai 2017 Late	69.5	73.1	86.9	6.8	19.8	0.87	0.65	72.2	4.3	0.98
Mean				7.2	17.5	0.86	0.69		3.7	0.98

Two cross-validation schemes were implemented for mimicking realistic prediction scenarios: CV0 corresponds to the scenario of predicting tested genotypes in unobserved environments; CV00 considers the prediction of untested genotypes in unobserved environments. For CV0, the C method was used combining phenotypic and day length information (DL) of genotypes tested in other environments (one at a time). For CV00, two methods were considered: (i) the conventional GS implementation; and (ii) the CB method, which combines phenotypic and DL information from tested genotypes (training set) and genomic BLUP values $\left( {\hat{g}} \right)$ from untested genotypes (testing set). In both cases, the prediction procedure was conducted by leaving one genotype out across environments and by deleting all phenotypic information from the target environment.

The C method was implemented under the CV0 scheme and their results were used for assessing the predictive ability of predicting an already tested genotype in an untested environment by considering only the phenotypic and daily DL information of the same genotype but observed in other environments. Across environments, this method returned an RMSE of 3.9 days and a PC of 0.98; within environments, the average RMSE and PC were 3.7 days and 0.98. The EMD ranged between -4.95 and 4.67 days.

**Figure 1:**
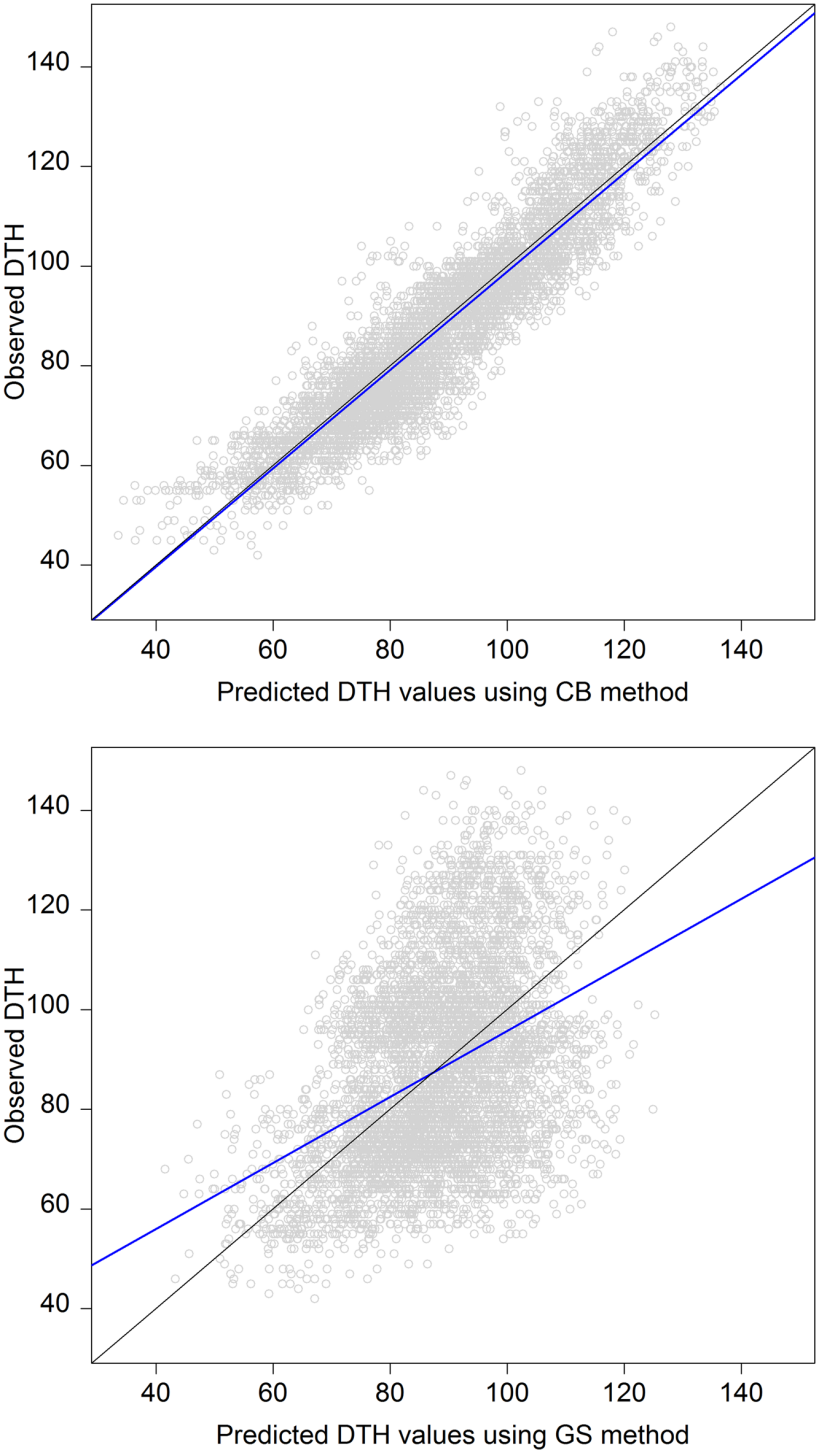
Scatter plot of predicted (*x-axis*) and observed (*y-axis*) DTH values under CB (top) and GS (bottom) methods for the CV00 scheme (predicting untested genotypes in unobserved environments) for a rice data set comprising 112 genotypes tested in 51 environments in Japan between 2005 and 2017. The CB method combines the predicted environmental means (E-mean) and the genomic BLUPs $\left( {\hat{g}} \right)$ derived from the conventional GS implementation while the GS implementation considers a common mean across environments plus the genomic BLUPs. The blue line represents the fitted line between predicted and observed DTH values, and the black line shows the exact match (on target) of the predicted values on the observed values.

The results of CB and GS methods are directly comparable between them (CV00 scheme). Figure 1 depicts the scatter plot between predicted and observed DTH values for all genotypes across environments. The top panel contains the results obtained with the proposed method (CB), while the bottom panel shows the results derived from the conventional GS implementation. The blue and black lines correspond to the fitted and the on target predictions with respect to the observed DTH values, respectively. In general, across-environments (Fig. 1) and within environments (Table 1) the results obtained with the CB method showed a better fit than those derived from the GS implementation.

**Figure 2:**
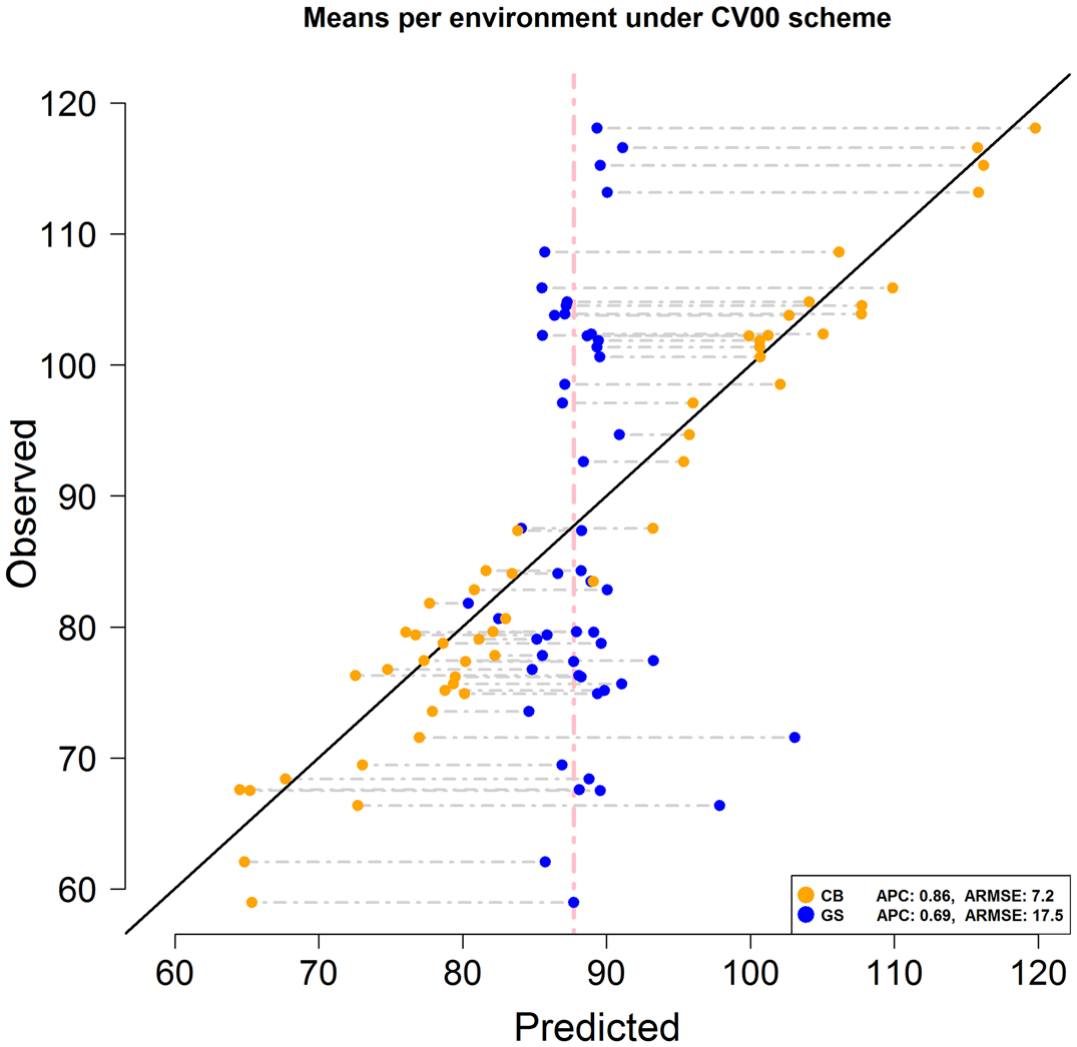
Scatter plot of predicted and observed environmental means (E-means) using the CB method (orange) and the conventional GS implementation (blue) for the CV00 scheme (predicting untested genotypes in unobserved environments) for a rice data set comprising 112 genotypes tested in 51 environments in Japan between 2005 and 2017. The vertical pink line indicates the position of the overall phenotypic mean across environments (88 days). The diagonal black line shows the exact match between predicted and observed means. ARMSE and ACP stand for average Root-mean-square error and average (across environments) Person correlation between predicted and observed DTH values. The RMSE and PC were first computed within environments and the obtained values were averaged.

The advantages of the CB method for predicting DTH are clearly appreciated when analyzing the predicted environmental means (E-means). Figure 2 displays the predicted (*x-axis*) and observed (*y-axis*) E-means under the CB (orange) and GS (blue) methods. As expected, when no information from the target environment was available the conventional GS implementation predicted DTH values centered on the overall mean (~ 88 days) of the environments in the training set. On the other hand, the CB method consistently returned values that were closer to the diagonal black line than those from the GS method. The black line represents the exact match between predicted and observed values.

## Discussion

The box-plot (Supplementary Fig. S1) showed a high heterogeneity between the environments of this study with those with a “late” planting date exhibiting smaller phenotypic variability and shorter occurrence time for DTH compared with the “early” planting environments. Among other considerations, extended (allowing the inclusion of the main effect of the environments) GS models assume homogeneity of variances between environments. However, the violation of this assumption may induce serious inconsistencies in the results, especially when the genotype-by-environment interaction parameters are not explicitly included in models.

The results from the GS model provide a clear idea of the problems we might incur predicting time-related traits when the phenotypic information from the target environment is not available (CV00). This cross-validation scheme represents among the four prediction scenarios that breeders face in field (CV2: predicting tested genotypes in observed environments; CV1: predicting untested genotypes in observed environments; CV0: predicting tested genotypes in unobserved environments; and CV00: predicting untested genotypes in unobserved environments) the most interesting, useful and challenging. Dealing with traits where the selection process is based on ranks rather than in the per se estimated values, the conventional implementation (GS) usually provides good results (moderate to high correlations) under the four schemes.

However, in traits related with important growth stages like DTH where the per se estimated values are crucial for designing, planning and establishing future experiments (i.e., CV0 and CV00 situations), the implementation of the conventional implementations (GS) might not be the best choice. When no phenotypic information from the target environment is available, the E-mean of the target environment is determined as the weighted average of those environments in the training set forcing it to be centered around a general mean which corresponds to the weighted mean of those environments in the training set. Hence, the final prediction is composed of this general weighted mean plus deviations corresponding to the estimated/predicted genomic effects. Since these deviations are conceptualized to be centered around zero, under CV0 and CV00 schemes the GS method would produce similar environmental means (i.e., centered on an overall mean determined by those environments in the calibration set).

The results from the C method showed that this implementation provides good estimations of DTH under the CV0 scheme. Across environments, the RMSE was of 3.9 days and a PC of 0.98. These results were similar to those obtained using the conventional GS models when the phenotypic information of the target environment was available (i.e., CV2 scheme) for other genotypes except for the one being predicted. Under CV2, the RMSE and Pearson correlation across environment were of 3.8 days and 0.98, respectively (data not shown).

As discussed before, it is expected to obtain similar environmental means using either CV0 or CV00 schemes under the conventional GS implementations. Under the CV00 scheme, across-environments the GS strategy returned a RMSE of 18.1 and a PC of 0.41. The EMD between predicted and observed values ranged between -31.5 and 28.7 days. On the other hand, under the CV0 scheme the C method returned EMD ranging between -4.95 and 4.67 days. In this case, the phenotypic information of those genotypes to be predicted in unobserved environments but tested in others was used. Since under the CV0 and CV00 schemes (i.e., when phenotypic information of the target environment is not available for any genotype) the conventional GS implementation produce similar E-means thus it is also expected that the proposed C method will produce better results than those derived from the GS implementation under the CV0 scheme.

The main goal of this research was to develop a prediction method able to deliver a small RMSE between predicted and observed values predicting DTH of untested genotypes in unobserved environments (CV00 scheme). Under this scenario, the CB method produced encouraging results. The across-environments RMSE was of 7.3 days for a PC of 0.86. A significant improvement was achieved compared with those results obtained using the conventional GS implementation (RMSE of 18.1 days and PC of 0.41). In this case, the CB method doubled the accuracy of the conventional GS method and reduced the RMSE around 60%. The advantages of the CB method are better appreciated comparing the predicted environmental means. Under the conventional GS implementation, the differences between the predicted and the observed environmental means ranged between -31.5 and 28.7 days while with the CB method these range between -6.4 and 4.1 days. The CB method reduced the extremes (left and right) of the GS range between 80 and 85%, respectively. Figure 2 shows that the CB environmental means (orange dots) were always closer to the diagonal line that represents the exact match with the true environmental means in comparison with those means derived from the conventional GS implementation (blue dots).

At the beginning the implementation of the C and CB methods requires enough information to accurately characterize the genotype’s DL curves and for this, sets of genotypes tested in many environments are necessary. However, once a good characterization of the DL curves is accomplished, potentially any untested genotype may be accurately predicted in any unobserved environment. Thus, similar results can be expected for a different set of genotypes and environments than those analyzed in this study.

The methods here introduced (C and CB) showed that the use of DL information significantly improves the predictive ability of the conventional GS implementations with an important reduction of the RSME. An advantage of the C method is that if the phenotypic information of the same genotype is available for a set of environments, the expected DTH for an unobserved environment can be easily estimated without requiring molecular marker information or phenotypic information from other genotypes. The CB method improved the predictive ability of untested genotypes in unobserved environments by coupling phenotypic and molecular marker information with the DL.

The introduced methods require the daily day length (DL) information of the environments (training and testing) in their design. The convenience of these two methods resides in the fact that the theoretical daily DL information can be obtained in advance before establishing and designing the experiments. Hence, the day length data potentially can help breeders to better schedule the crop season. The proposed methods capture the genotype-by-environment interaction (G × E) component via the fitted curves thus the explicit inclusion of this term is not needed. Moreover, the conventional GS implementation returns the same genomic estimated effect across environments for a particular genotype when the G × E component is absent. The curves precisely capture the sensitivity experienced by genotypes under different environments driving specific responses for each environment. Although the CB method is composed of the genomic component computed without explicitly considering the G × E term, the final prediction accounts for this interaction term via the curves. In addition, the formal modeling of the heterogeneity of variances is not necessary because these patterns are also accounted for the proposed method via the curves.

## Materials and methods

### Phenotypic data

The phenotypic data set comprises the seeding and heading date of 112 Japanese rice cultivars with different origin from different regions and these correspond to improved lines and landraces (Supplementary Table S1). The genotypes were tested in 51 locations (North to South of Japan) from 2004 to 2017 (Supplementary Table S2). This multi-environmental trial (MET) experiment was conducted for investigating heading date related genes. The materials have polymorphisms known as heading date related genes (e.g., Hd1, Hd2, Hd5, and Hd6).

The number of DTH was calculated as the number of days that occur between the heading date and the seeding date. The heading date was determined when more than 50% of the individuals in the plot reached the heading stage.

### Marker genotype data

A detailed description of the DNA extraction procedure and library construction of the whole-genome shotgun sequence can be found in Yabe et al.^16^*.* For each variety, total DNA was extracted using the CTAB method^17^. The library was constructed using the Illumina TruSeq DNA Sample preparation kit according to the manufacturer's instructions (Illumina, Inc., San Diego, CA, USA). Next-generation sequencing data was obtained using the Illumina HiSeq 2000, HiSeq 4000 and HiseqX systems via paired-end sequencing (Illumina, Inc., San Diego, CA, USA). The sequence data have been deposited in the DNA Data Bank of Japan (DDBJ) Sequence Read Archive, under Submission DRA008071. Data sets deposited in the DDBJ Sequence Read Archive (SRA106223, ERA358140, DRA000158, DRA000307, DRA000897, DRA000927, DRA007273, DRA007256) were reanalyzed. Adapters and low-quality bases were removed from paired reads using the Trimmomatic v0.36 program^18^. The preprocessed reads were mapped to the Os-Nipponbare-Reference-IRGSP-1.0^19^ by using the bwa-0.7.12 mem algorithm with the default options^20^. The mean depth of the reads against the Os-Nipponbare-Reference-IRGSP-1.0 ranged from 7.4× to 72.4× with an average of 16.9×.

SNP calling was based on alignment using the Genome Analysis Toolkit^21^ (GATK, 3.7-0-gcfedb67) and Picard package V2.5.0 (https://broadinstitute.github.io/picard). The mapped reads were realigned using RealignerTargetCreator and indelRealigner of GATK software. SNPs and InDel were called at the population level using the UnifiedGenotyper of GATK with the -glm BOTH option. Heterozygous loci were treated as missing, owing to the assumption that almost all markers were well fixed in rice cultivars after several generations of self-pollination. We used only biallelic non-missing sites overall cultivars with a minor allele frequency (MAF) ≥ 0.025. Initially, 629,602 SNP markers were considered in the study. After applying conventional quality control (i.e., discarding those SNPs with more than 50% of missing values and a MAF smaller than 0.03) 408,372 SNPs remaining in the study for analysis.

### Day length data

Although there are several definitions for day length (DL) according to the Smithsonian Institution^22^ roughly speaking, it can be defined as the amount of time in a day with sunlight. The theoretical DL values were computed by the default method^23^ included in the geosphere (v1.5-5) R package^24^. DL information can be known in advance just by knowing the location and date, and it is not influenced by the weather conditions.

### Cross-validation schemes

Two cross-validation schemes were considered in this study and these mimic different situations that breeders might face in fields. Supplementary Fig. S2 shows a graphical representation of CV0 (predicting tested genotypes in unobserved environments) and CV00 (predicting untested genotypes in unobserved environments). For CV0 (top panel in Supplementary Fig. S2), the cross-validation is conducted by deleting the phenotypic information from all genotypes at each environment (one at a time). Then, the remaining environments are used as training set.

For implementing the CV00 scheme, not only the phenotypic information of the testing environment is deleted (same as in the previous cross-validation scenario) but also the phenotypic information of the genotype to be predicted is deleted from all environments (one at a time). In the bottom panel of Supplementary Fig. S2, we have that the goal was to predict genotype G3 in environment E4. For this environment, the same procedure is repeated for each one of the genotypes (G1–G5). Such that with six environments and five genotypes we have that this procedure is implemented 30 (5 × 6) times considering the toy example displayed in this panel (bottom).

### GBLUP model

The Genomic Best Linear Unbiased Predictor model (Genomic BLUP or GBLUP) is the most common and convenient main effects model used in GS applications^8,25^. It attempts to explain DTH of the *i*th genotype (*i* = 1, 2,…, *I*) observed in the *j*th environment (*j* = 1, 2, …, *J*) as the sum of a common mean plus random environmental and genomic effects, and an error term, and it is represented as follows:

1 $$Y_{ij} = \mu + E_{j} + g_{i} + e_{ij} ,$$

where $$\mu$$ is a constant effect common to all genotypes across all environments;$$E_{j}$$ are IID (independent and identically distributed) outcomes from a normal distribution $$N\left( {0,\sigma_{E}^{2} } \right)$$ representing the effect of the *j*th environment; $$g = \left\{ {g_{i} } \right\}\sim N\left( {0,{\text{G}}\sigma_{g}^{2} } \right)$$ is the vector of genomic effects, with $${\text{G}} = \frac{{{\text{XX}}^{\prime } }}{p}$$ as the genomic relationship matrix whose entries describe the genetic similarities between pairs of individuals, $${\text{X}}$$ is the standardized (by columns) matrix of $$p$$ molecular markers; $$\varepsilon_{ij}$$ are IID outcomes from a $$N\left( {0,\sigma_{e}^{2} } \right)$$ representing the error term; and $$\sigma_{E}^{2} ,$$$$\sigma_{g}^{2}$$ and $$\sigma_{e}^{2}$$ are the corresponding associated variance components of the described random effects.

Usually, when METs are analyzed the environmental component explains the larger proportion of the phenotypic variability across environments^26,27^. Hence, from (1) it can be concluded that the predicted DTH of the *i*th genotype in the *j*th environment strongly depends on the environmental mean rather than on the genomic component. This environmental mean is composed by a common mean effect (across environments) plus a deviation (around zero) that corresponds to the effect of *j*th environment.

Conceptually, the common mean effect can be viewed as the average performance of all genotypes across environments. The environmental deviation corresponds to the mean effect of the current environmental *stimuli* in the set of the tested genotypes beyond the common mean. Thus, the environmental effects can be considered as deviations around the common mean. Hence, by construction, the contribution of the genomic component to the idealized phenotype is much smaller in comparison with the overall mean and the environmental effects.

If the phenotypic information (DTH) of the target environment is available for a set of already observed genotypes, good approximations of the environmental effects can be obtained. Otherwise, the environmental effects are estimated using the phenotypic information of those genotypes observed in other environments. In this case, the environmental component is estimated as the weighted average of the environmental effects in the training set. Since these random effects are conceptualized to be centered on zero, the weighted average will be close to zero. Hence, a predicted DTH value will be mainly composed of a common mean and a small genomic effect. This would shrink the predicted values towards the common mean. Although this method usually delivers high correlations between predicted and observed values, it also induces a large bias (RMSE) when predicting time-related traits.

To alleviate this, the two introduced methods (C and CB) attempts to leverage the DL information in the prediction process by considering the curves of the daily day length in the prediction process. The idea for using DL information in the prediction process was motivated when examining the relationship between the Cumulative Day Length (CDL) and DTH for all genotypes across environments (Supplementary Fig. S3). A simple linear regression of DTH on CDL returned an R-squared value of 0.99, exhibiting a strong relationship between these two variables. We can conclude that if the CDL values are known then accurate estimations of DTH may be obtained for any genotype in any environment. However, in order to compute the CDL, the exact number of days between the planting date and the heading occurrence should be known first. This implies that DTH should be known beforehand, which makes unfeasible the direct use of CDL for prediction purposes. However, these results provide strong evidence about the potential of the DL information for improving the prediction accuracy of DTH.

**Figure 3:**
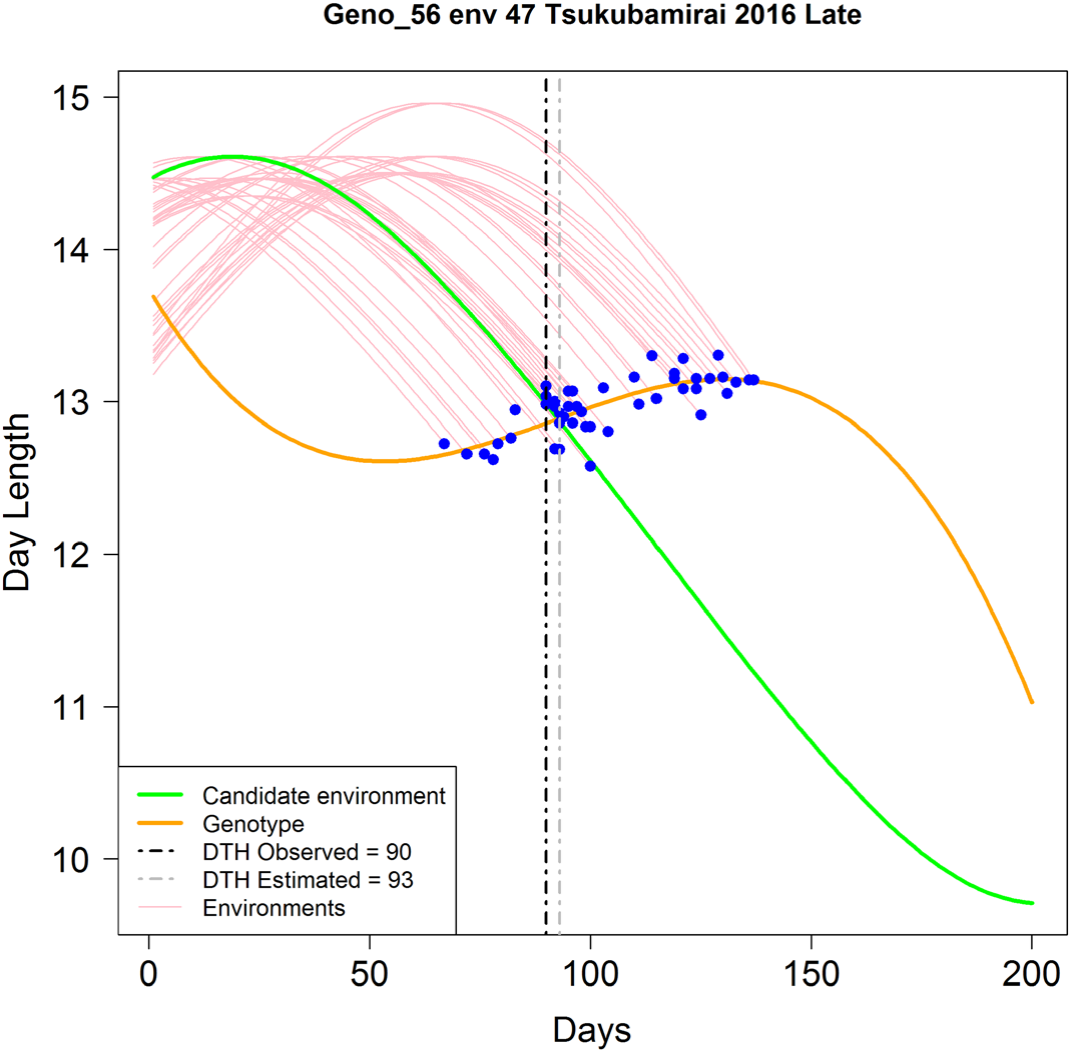
Graphical representation of the C method for estimating days to heading (DTH, *x-axis*) of genotype 56 in environment 47 (env 47 Tsukubamirai 2016 Late). Pink curves represent the daily day length (hours) values (DL, *y-axis*) from planting day (day 1) until heading time for genotype 56 in 50 environments (1–46, and 48–51). The blue dots indicate the corresponding DL values when genotype 56 reached heading time. The green curve (C_2_) represents daily DL progression of the target environment (Environment 47) where no genotypes have been observed yet. The orange curve (C_1_) represents the fitted line for genotype 56 using a third-degree polynomial equation relating DL at the occurrence time (DTH across environments). The vertical dotted gray line shows the intersection (93 days) between the green and the orange curves (C_1_, C_2_). This value is the estimated DTH for genotype 56 in the unobserved environment “env 47 Tsukubamirai 2016 Late”. The vertical dotted black line corresponds to the actual DTH (90 days) value for env 47.

Since each genotype was observed in a considerable number of environments (between 48 and 50), it is possible to find specific response patterns between DTH and DL analyzing each genotype at a time. In this study, across environments, we observed that the DL values at the DTH occurrence present strong response patterns with DTH. For illustrating the conceptualization of this idea, we use the example depicted in Fig. 3. It corresponds to phenotypic (DTH) and DL information of genotype 56 observed in 50 environments out of 51 (observed in environments 1–46, and 48–51 but not in env 47). The goal in this example is to estimate DTH for this genotype in the unobserved environment (env 47: Tsukubamirai 2016 Late). For each environment, the planting date was considered as day 1 (*x-axis*) then the daily DL (*y-axis*) values were plotted (pink lines) from day 1 (planting) until the time when genotype 56 reached DTH (blue dots). In Fig. 3, a pattern relating DTH and DL at the occurrence day is easily identified. This gradient seems not to be strongly affected by the initial DL values at the different planting dates across environments. However, it appears to be linked with the initial DL’s changing rate at the planting day. Those environments where DL’s changing rates decreased at the planting time (late cultivars) showed the shortest DTH values and vice-versa.

For CV00 scheme, the proposed method requires: (i) first the estimation of DTH in a target/unobserved environment (CV0) for a set of already tested (training) genotypes (one at a time) yet to be observed; (ii) then compute the expected environmental mean of the unobserved environment using the estimated values derived from the previous step (i); (iii) the final step consists of adding to the estimated environmental mean the resulting genomic BLUPs of the untested genotypes derived from the conventional GS method. Further details of these steps are given below.

### C method

The DTH estimation of a tested genotype in an unobserved environment (CV0) requires phenotypic and DL information across several environments. The first step considers the characterization of DL at the occurrence of heading time across environments. For this, a third-degree polynomial equation is used to model DL as a function of DTH (orange curve in Fig. 3) as follows:

2 $$dl_{ij} = b_{0} + b_{1} t + b_{2} t^{2} + b_{3} t^{3} + \varepsilon_{ij} ,$$

where $dl_{ij}$ represents the observed DL of the *i*th genotype in the *j*th environment; $t$ is the time independent variable; $b_{0}$, $b_{1}$, $b_{2}$, and $b_{3}$ are the model coefficients; and $\varepsilon_{ij}$ is the error term and it is assumed to be IID $N\left( {0,\sigma_{\varepsilon }^{2} } \right)$. This model is referred as C_1_.

Similarly, the daily DL information of the target environment (green curve) is modeled using a third-degree polynomial equation:

3 $$E_{j} = b_{E0} + b_{E1} t + b_{E2} t^{2} + b_{E3} t^{3} + \xi_{ij} ,$$

with $b_{0E}$, $b_{1E}$, $b_{2E}$, and $b_{3E}$ correspondent to the model coefficients; and $\xi_{ij}$ is the error term and it is assumed to be IID $N\left( {0,\sigma_{\xi }^{2} } \right)$. This model is referred as C_2_. The solution to this system of two equations (C_1_, C_2_) will produce an estimate of DTH in the target environment for the genotype under analysis only (genotype 56 in this case). In order to find the intersection of these two curves, a numerical evaluation was implemented. In this example, the solution was found to be 93 days to heading (Fig. 3; vertical dotted gray line) while the observed DTH was of 90 days.

### CB method

Since the ultimate goal is to predict DTH for untested genotypes in unobserved environments, the first step is to estimate the expected DTH mean of the target environment. Then, the final prediction is composed of the expected DTH mean of the target environment plus the genomic BLUP ($\hat{g}_{i}$) of the untested genotype obtained via the conventional GS implementation. For estimating the DTH mean, the C method was first implemented in a set of already tested genotypes (training set) for predicting their corresponding DTH on the target environment. Here, only the phenotypic and DL information of those genotypes in the training set was considered and the predicted values were averaged.

**Figure 4:**
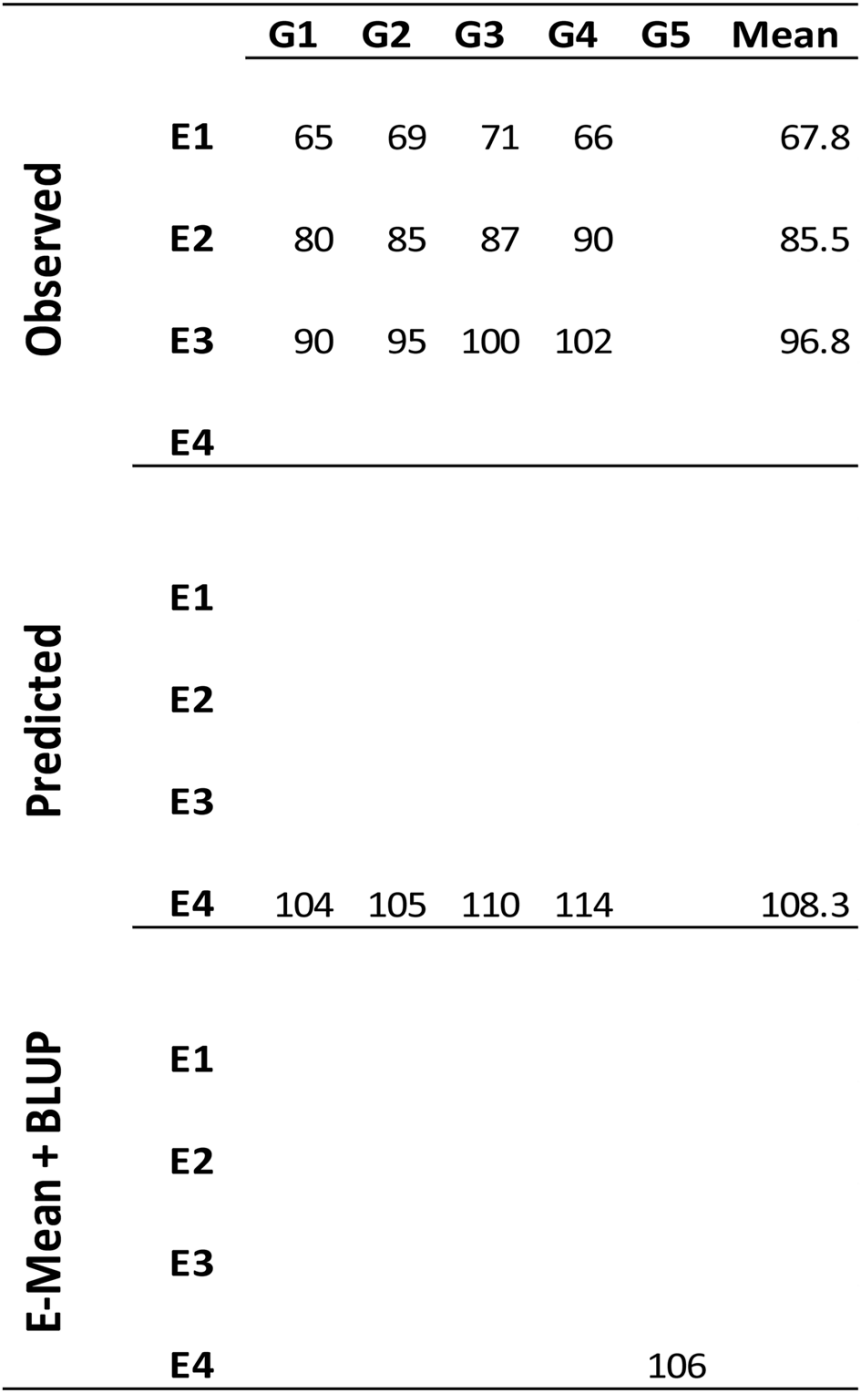
Representation of the procedure for predicting DTH for the untested genotype G5 in the unobserved environment E4 using the information of four genotypes (G1, G2, G3, and G4) observed in three environments (E1, E2, and E3). The top panel contains the observed values for genotypes (G1, G2, G3, and G4) in environments (E1, E2, and E3). The middle panel contains the predicted values that presumably were obtained with the C method for these genotypes in the unobserved environment E4. Also, the E-mean (108.3) of E4 is computed as the mean of the predicted values for genotypes G1, G2, G3, and G4. The bottom panel contains the predicted DTH value (106) of genotype G5 in environment E4 as the sum of the E-mean (108.3) computed in the previous step and the genomic BLUP (-2.3) obtained with the conventional GS implementation.

Figure 4 shows the process for predicting untested genotypes in unobserved environments using a toy example consisting of five genotypes (G1, G2, G3, G4, and G5) and four environments (E1, E2, E3, and E4). In this example, we describe the procedure for predicting DTH of the untested genotype G5 in an unobserved environment (E4). The top panel of Fig. 4 contains the observed values of genotypes 1–4 in environments 1–3. The middle panel shows the predicted values presumably obtained with the C method for genotypes 1–4 (104, 105, 110, and 114) in E4, the unobserved environment. Also, the predicted E-mean (108.3) was computed there as the mean of the predicted DTH values. The bottom panel shows the predicted DTH for genotype G_5_ in the environment E4 (106). This value (106 = 108.3-2.3) was obtained as the sum of the E-mean and the genomic BLUP value (-2.3) obtained for genotype G5 using the conventional GS implementation.

### Software

The C and CB methods, as well as all the statistical analyses were performed using the R-software R Core Team^28^. The conventional GS model was fitted using the Bayesian Generalized Linear Regression (BGLR) R-package^29,30^.

## Supplementary information

Supplementary Information.

## Data Availability

Genome data can be accessed from DDBJ under submission DRA008071.
