# Supplementary material for: Coupling day length data and genomic prediction tools for predicting time-related traits under complex scenarios
Source: Sci Rep. 2020 Aug 7;10:13382. doi: 10.1038/s41598-020-70267-9 (PMC7415153; doi:10.1038/s41598-020-70267-9)
Supplement: Supplementary file 1 — Supplementary Information. [file 41598_2020_70267_MOESM1_ESM.docx]

**Coupling Day Length Data and Genomic Prediction tools for Predicting Time-Related Traits under Complex Scenarios**

**Supplementary Materials**

Diego Jarquin^¥,*^, Hiromi Kajiya-Kanegae^†^, Chen Taishen^†^, Shiori Yabe^‡^, Reyna Persa^¥^, Jianming Yu^**^, Hiroshi Nakagawa^a^, Masanori Yamasaki^§^, Hiroyoshi Iwata^†,*^

^¥^Department of Agronomy and Horticulture, University of Nebraska – Lincoln, Lincoln NE, 68583, USA.

^†^Graduate School of Agricultural and Life Sciences, The University of Tokyo, Bunkyo, Tokyo 113-8657, Japan.

^‡^Institute of Crop Sciences, National Agriculture and Food Research Organization (NARO), Tsukuba, Ibaraki 305-8518, Japan

^**^Department of Agronomy, Iowa State University, Ames, USA.

^§^Food Resources Education and Research Center, Graduate School of Agricultural Science, Kobe University, Kasai, Hyogo 675-2103, Japan

^a^Institute for Agro-Environmental Sciences, National Agriculture and Food Research Organization (NARO), Tsukuba Ibaraki 305-8604

**Abstract**

This file contains the supplementary figures and tables for the main text.

**Supplementary Figures**

**
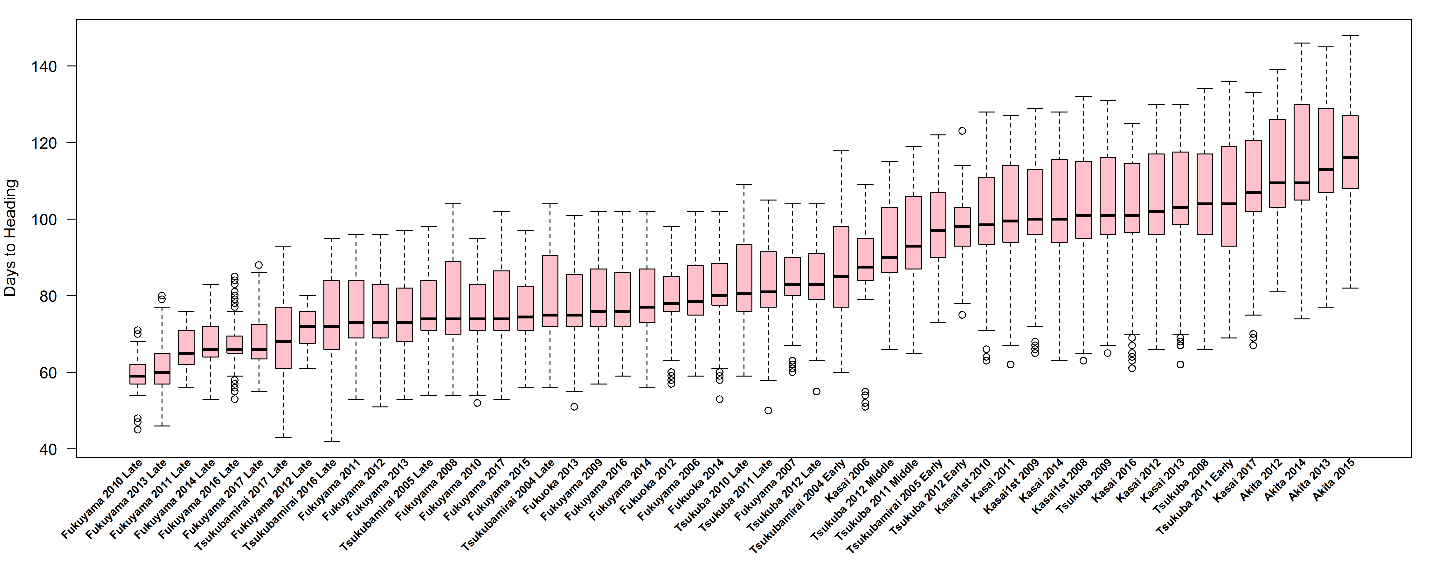
**

**1.** Figure S1. Box-plot for days to heading (DTH) for a rice data set comprising 112 genotypes tested in 51 environments in Japan between 2005 and 2017. From the total of all possible genotype-environment combinations (112×51=5712), 94% (5365) of these were observed. The environments were ordered based on their environmental medians.

**2**. Figure S2. Graphical representation of the cross-validation schemes CV0 (top) and CV00 (bottom) using five genotypes and six environments. The CV0 scheme mimics the prediction scenario of predicting tested genotypes in unobserved environments (E4). This scheme is implemented leaving one environment out at a time and using the remaining as training set. The CV00 scheme mimics the prediction scenario of predicting untested genotypes (G4) in unobserved environments (E4). This scheme is implemented leaving one environment out at a time and one genotype (across environments) out at a time and using the remaining environments and genotypes as training set.


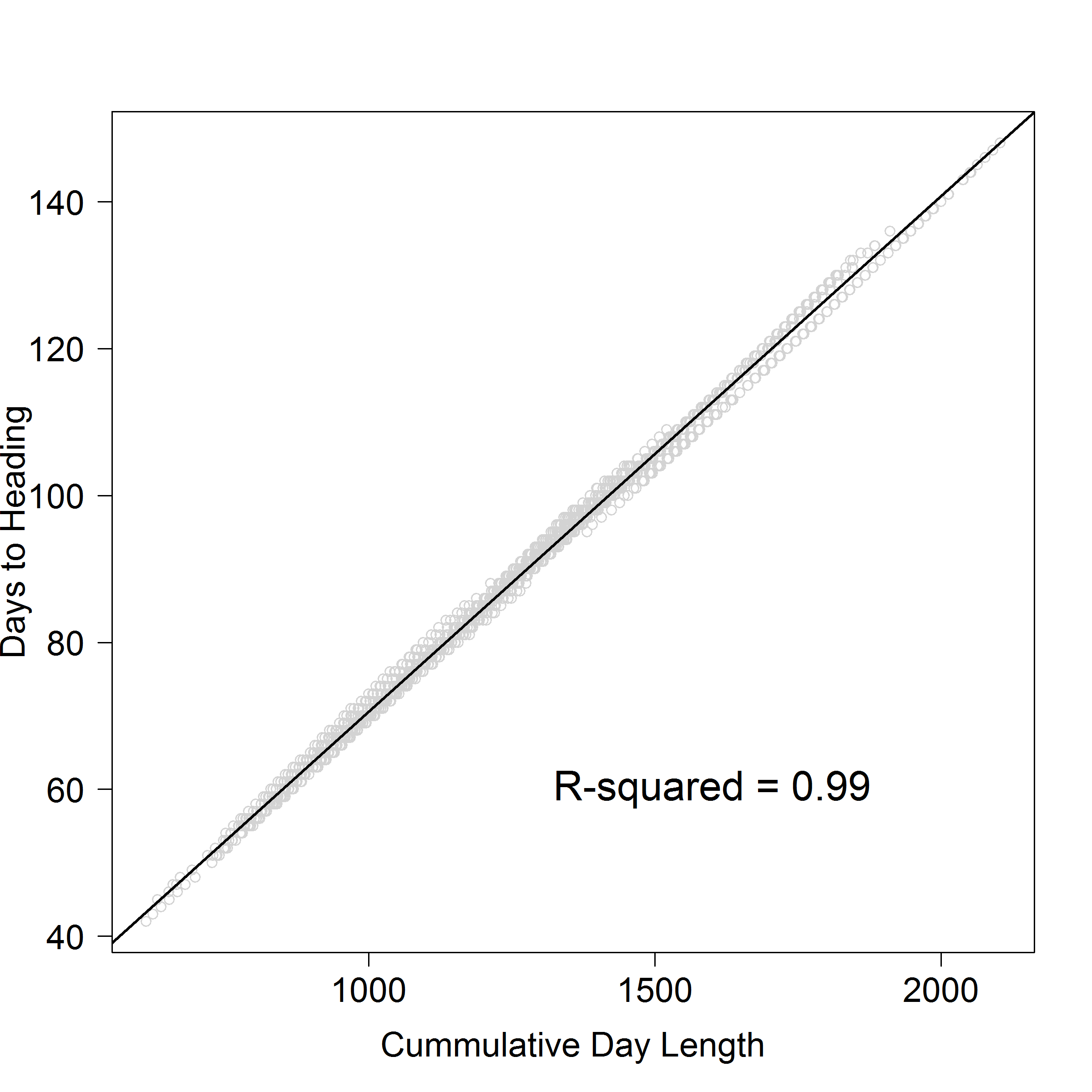


**3.** Figure S3. Scatter plot of days to heading (DTH) as a function of the cumulative day length (CDL) from planting date until the occurrence event for a rice data set comprising 112 genotypes observed in 51 environments (in Japan between 2005 and 2017). From the total of all possible genotype-environment combinations (112×51=5712), 94% (5365) of these were observed. The black line represents the regression line between DTH and CDL (R-squared of 0.99).

**Supplementary Tables**

**4.** Table S1. The 112 Japanese rice cultivars used in this study with pedigree and region of origin.

**5.** Table S2. Rice trials for studying days to heading for 112 genotypes tested in 79 environments (site-by-year-by-planting date combinations) in multiple locations in Japan. In locations with more than 1 experiment per year, different seeding times were tested.
